# The Impact of Organic Micropollutants on the Biochemical Composition and Stress Markers in *Wolffia arrhiza*

**DOI:** 10.3390/molecules30030445

**Published:** 2025-01-21

**Authors:** Urszula Kotowska, Alicja Piotrowska-Niczyporuk, Justyna Kapelewska, Lilla Lane Jasinska

**Affiliations:** 1 Department of Analytical and Inorganic Chemistry, Faculty of Chemistry, University of Bialystok, Ciolkowskiego 1K Str., 15-245 Bialystok, Poland; j.kapelewska@uwb.edu.pl (J.K.); llanejasinska@sierracollege.edu (L.L.J.); 2 Department of Plant Biology and Ecology, Faculty of Biology, University of Bialystok, Ciolkowskiego 1J Str. 15-245 Bialystok, Poland; alicjap@uwb.edu.pl; 3 Department of Chemistry, Sciences and Mathematics Division, Sierra College, 5100 Sierra College Blvd, Rocklin, CA 95677, USA

**Keywords:** personal care products, industrial chemicals, pesticides, photosynthetic pigments, proteins, monosaccharides, hydrogen peroxide, malondialdehyde

## Abstract

For many years, there has been a growing pollution of the aquatic environment with personal care products and industrial chemicals, the main source of which is municipal and industrial wastewater. This raises the need to assess the impact of these pollutants on ecosystems, including plants living in the aquatic environment. It is important to develop methods for their removal from wastewater, among which using plants for phytoremediation is a promising solution. This study aimed to evaluate the response of the aquatic plant *Wolffia arrhiza* (Lemnaceae) to low concentrations of bisphenol A (BPA), *N*,*N*-diethyl-*m*-toluamide (DEET), triclosan (TRC), benzophenone (BPH), endosulfan alpha (α-END), and endosulfan beta (β-END). The plant growth, the content of cellular components, and oxidative stress markers were assessed in response to plant contact with single compounds at concentrations of 0.1 mg/L and 1 mg/L, and their mixture at a total concentration of 1 mg/L. All of the pollutants used in the study inhibited the *W. arrhiza* growth and stimulated the degradation of proteins but enhanced the level of saccharides. TRC, BPH, α-END, and β-END had a negative impact on the content of photosynthetic pigments. Increased concentrations of the oxidative stress markers MDA and H_2_O_2_ were registered in the plants exposed to BPA, TRC, and β-END. The mixture of pollutants had higher toxic effects than individual substances.

## 1. Introduction

In the era of continuous industrial development and changes in the way societies function, substances with properties that adversely affect living organisms have appeared in the water, including compounds classified as organic micropollutants (OMPs). These are compounds of both natural and anthropogenic origin, the concentrations of which in the environment range from nanograms to micrograms per liter [1]. The literature shows that, in surface waters, the tested compounds ranged from several ng/L to several μg/L [2,3,4]. In surface water, the largest amounts were determined for BPA (<798 ng/L) [3], while the smallest content was determined for endosulfans (below 50 ng/L) [4]. So far, no α-END and β-END determinations have been performed in wastewaters and groundwaters. The remaining contaminants in the abovementioned matrices occur at levels of <12.06 μg/L and below 17.3 μg/L, respectively [3,5]. A significant part of these substances are referred to as contaminants of emerging concern (CEC) due to their suspected or confirmed negative impact on living organisms [6]. Most OMPs are unstable in the environment and are not resistant to physical, chemical, and biological factors. However, their constant presence in the environment is observed, and this apparent durability results from their continuous introduction into the environment [7]. The main source of OMPs is wastewater discharged into water and soil after insufficiently effective treatment processes [8]. At the same time, landfills or runoff from agriculture are significant sources of some of these substances [9]. In the case of many OMPs, there is a lack of sufficient data on the effects, fate, and concentration levels that would allow for conclusions to be drawn regarding the safety of their use. Therefore, legal regulations concerning these substances are only slowly being introduced into national and international legislation. An example is the update of the Wastewater Directive by the European Parliament, which imposes the obligation to monitor the presence and removal of OMPs by wastewater treatment plants, in a broad time perspective until 2045 [10].

Improving the efficiency of OMP removal in wastewater treatment processes is one of the most important contemporary challenges of environmental protection. The greatest attention is paid to physical methods (sorption, micro- and nanofiltration, and reverse osmosis) and chemical methods (advanced oxidation processes (AOPs)) [11,12,13,14]. These methods are usually highly effective, but their use is associated with additional challenges and threats. Physical methods involve transferring contaminants from one medium to another, and the disposal of heavily contaminated membranes or sorbents is very expensive. AOPs, on the other hand, apart from being associated with high costs, can lead to the formation of oxidation products that are equally or even more toxic than the initial contaminants. Biological methods using bacteria, fungi, or plants can, under appropriate conditions, be an equally effective and, at the same time, more economical and ecological solution [15]. Hydrophyte treatment, i.e., using aquatic and water-loving plants (hydrophytes) together with microorganisms coexisting with them to purify wastewater and other polluted waters is one of the types of plant-based remediation (phytoremediation) [16]. Rooted plants such as common reed (*Phragmites australis*), broadleaf cattail (*Typha latifolia*), yellow iris (*Iris pseudoacorus*), lake mole (*Schoenplectus lacustris*), branched coneflower (*Sparganium ramusom*), and manna mullet (*Glyceria aquatica*) can be used in hydrophyte treatment, which are highly effective but require a large area, appropriate soil conditions, and a long time to achieve the effect. Duckweed species (Lemnaceae) have gained considerable attention as effective organisms for the phytoremediation of polluted waters [17]. These small floating plants, widespread in all latitudes, are highly resistant to external conditions, including high concentrations of nitrogen, phosphorus, organic compounds, and heavy metals. Plants from the Lemnaceae family have low requirements. They are easy to grow, which can be performed in natural water reservoirs or indoors in bioreactors, allowing for the efficient use of space and vegetation in controlled and repeatable conditions [18]. The rapid course of life processes, reproduction, and the ability for mixotrophic nutrition result in the rapid uptake of inorganic and organic compounds from water [19]. The chemical composition of Lemnaceae plants (dry weight) is as follows: 18–35% of carbohydrates, 21–38% of starch, 16–42% of protein, and 4.5–9% of lipids, which make duckweed a possible feedstock for biomass-based energy operations (anaerobic digestion, incineration, pyrolysis, gasification, and oxidation), and the production of biofuels, biochars, and natural fertilizers through composting [20,21]. This opens the way to the development of plants after phytoremediation and obtaining new materials or energy from them.

*Wolffia arrhiza*, one of the 36 species belonging to the family Lemnaceae, is the smallest vascular plant in the world, commonly found in most freshwater ecosystems. It has a high capacity to bioaccumulate pollutants and, therefore, can be used in the phytoremediation of many inorganic and organic compounds. *Wolffia* is highly effective in removing arsenic and other heavy metals [22,23,24], nutrients [25,26], phthalates [27], benzotriazoles [28,29], and other OMPs [30] from water by biosorption, bioconcentration, and biodegradation. Understanding the nature and mechanism of the processes that are triggered when *W. arrhiza* comes into contact with contaminants present in water is important for the optimal planning of phytoremediation and expanding the application scope of this plant [29]. On the other hand, *W. arrhiza* is a good model organism for testing the effects of organic toxicants, as its growth, physiology, and biochemical responses have been extensively studied during the last few years [31,32]. Therefore, this floating plant is an excellent candidate for practical applications to indicate water contamination and in ecotoxicological studies. The micropollutants selected for research, such as BPA, DEET, TRC, BPH, α-END, and β-END (their characteristics are included in Table 1), are compounds with a proven negative impact on animals and humans, especially at developmental stages. The studies described in the literature indicate a potential negative influence of OMPs on plant growth and biochemistry [33]. However, the response of aquatic plants to low OMP concentrations is still unclear. There is also a lack of scientific data about the possible toxic effects of all of the mentioned OMPs on *W. arrhiza*, which plays an essential role in the aquatic environment. Thus, our study aimed to assess the effect of BPA, DEET, TRC, BPH, α-END, and β-END, as well as their mixture in low concentrations, similar to those found in wastewater and other polluted water, on plant growth, the contents of photosynthetic pigments, monosaccharides, and proteins, as well as the levels of oxidative stress markers. Therefore, the novelty of the current work is in evaluating the toxicity of different OMPs alone and the mixture of these compounds to *W. arrhiza* due to its widespread distribution and environmental relevance.

## 2. Results and Discussion

### 2.1. Plant Growth

**Figure 1:**
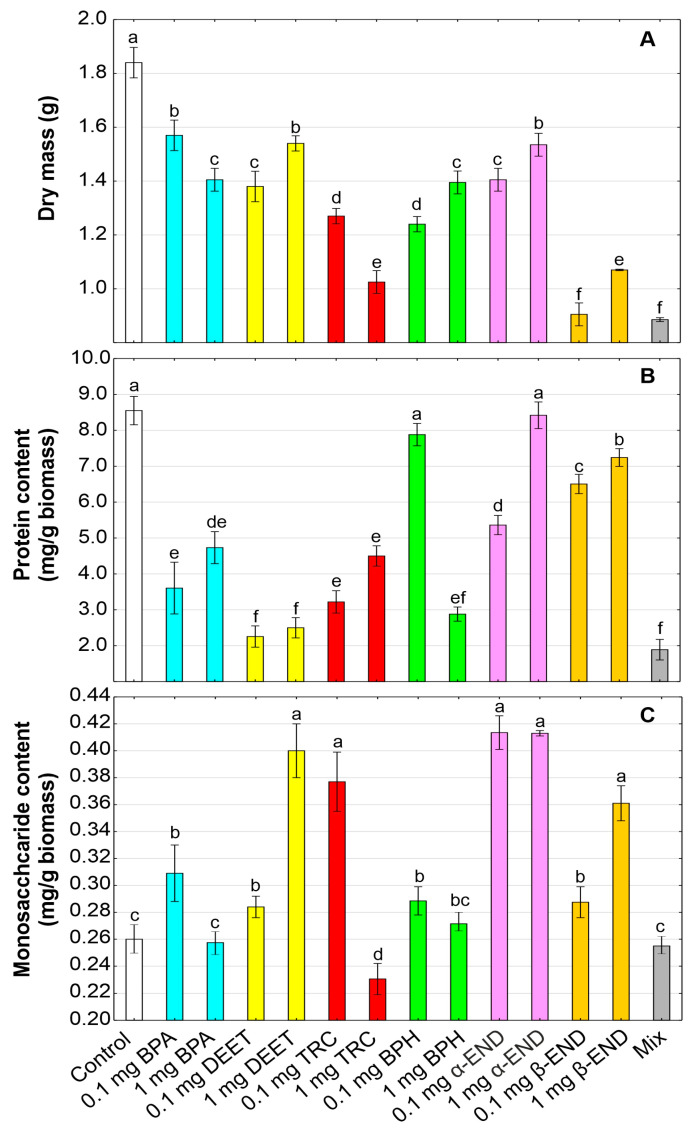
Dry mass (**A**), protein (**B**), and monosaccharide (**C**) contents in *Wolffia arrhiza* cultures under the influence of the following micropollutants: BPA (0.1 and 1 mg/L), DEET (0.1 and 1 mg/L), TRC (0.1 and 1 mg/L), BPH (0.1 and 1 mg/L), α-END (0.1 and 1 mg/L), and β-END (0.1 and 1 mg/L), as well as their mixture, in relation to control on the 7th day of culture. Data are the means of three independent experiments ± SD. Treatments with at least one letter being the same indicate a non-significant difference according to Tukey’s post hoc test.

In this study, the freshwater floating plant *W. arrhiza* was exposed to BPA (0.1 and 1 mg/L), DEET (0.1 and 1 mg/L), TRC (0.1 and 1 mg/L), BPH (0.1 and 1 mg/L), α-END (0.1 and 1 mg/L), and β-END (0.1 and 1 mg/L), and their mixture (the concentration of each compound at 0.167 mg/L and total the OMP concentration at 1 mg/L), with a control culture (Figure 1). The obtained results indicated that the mixture of all of the OMPs was characterized by the highest toxic effect on *W. arrhiza*, because the plant growth expressed as dry mass was inhibited by 51.9% compared to the control. Among all of the tested pollutants, β-END at a concentration of 0.1 mg/L was the most effective at inhibiting plant growth, by 50.8%, in relation to the control. The toxic effects on plant growth also characterized TRC, because the dry mass was reduced by 44.3% in the plants treated with 0.1 mg/L of TRC and 31.0% in plants exposed to 1 mg/L of TRC. The decreases by 14.7–23.6%, 16.3–25.0%, 24.2–32.6%, and by 16.6–23.6% were observed in plant cultures subjected to BPA (0.1 and 1 mg/L), DEET (0.1 and 1 mg/L), BPH (0.1 and 1 mg/L), and α-END (0.1 and 1 mg/L), respectively, in comparison with the control. Thus, exogenously applied OMPs such as BPA, DEET, BPH, and α-END possessed similar toxicological properties on plant growth and development.

Like other pesticides, endosulfan represents a strong inhibitory influence on plant growth. For example, *Phaseolus leptostachyus* plants subjected to endosulfan stress were characterized by a lower germination rate, a reduced root and stem length, the decreased fresh weight of the organs (root, stem, and leaf), and a lower leaf water content [39]. Thus, the obtained results on *W. arrhiza* confirmed the toxicity of α-END. Studies have also revealed the deleterious effects of endosulfan at lower concentrations on seed germination, plant growth, and nutrient uptake in tomato *Lycopersicum esculentum* [40,41], the diversity and presence of microorganisms in aquatic and land ecosystems [42,43], and health problems in animals [44]. The high toxicity of endosulfan and its related isomers in plants may be due to their high bioaccumulation rate, a long residual period in the environment, and long-distance transportation [45].

The harmful influence of TRC on *W. arrhiza* fronds may be connected with a high bioaccumulation rate, which was previously observed in the tissues of various plants, including vegetables and agricultural crops, e.g., carrot, pumpkin, lettuce, cabbage, radish, soybean, pepper, cucumber, and peanuts [46,47]. The uptake of organic pollutants, such as TRC, may consequently affect the growth and development of plants. Because of the wide distribution of TRC in aquatic and terrestrial environments, and the possibility for the easy potential entry into the food chain, this compound possesses high risks to human health. Our results were confirmed by other studies performed on wetland macrophytes (*Sesbania herbacea* and *Bidens frondosa*) exposed to TRC at concentrations at a range of 0.4–1.000 ppb. TRC application reduced the seed germination, total fresh weight, shoot and root fresh weights, root length, and root surface in these aquatic plants [48]. Moreover, the authors of [49] showed that the toxicity of this bacteriostatic agent substance depends on the plant species used in the experiments. For example, the inhibitory influence of TRC on the submerged plant *Hydrilla verticillata* was significantly higher than that on the floating plant *Eichhornia crassipes*. Probably, *H. verticillata* possesses higher biosorption properties, specific growth characteristics, and a greater sensitivity to this organic compound [49].

Organic micropollutants, such as BPA, DEET, BPH, and α-END, were characterized by lower harmful effects on *W. arrhiza* growth than β-END and TRC. Similarly, BPH was reported to be less toxic to invertebrates (e.g., *Dugesia japonica* and *Daphnia magna*), fish, and the green microalga *Chlamydomonas reinhardtii* based on the toxicological parameters, such as the EC50 and the lowest-observed-adverse-effect level [50,51]. Additionally, previous studies indicated that BPA elicited slight-to-moderate toxicity depending on the concentrations and the plant species used in the experiments [52]. For example, the treatment with BPA at low doses of <3 mg/L was beneficial for the growth, development, and antioxidant defense system in *Oryza sativa* [52,53]. On the other hand, radish (*Raphanus sativus*) seeds subjected to various concentrations of DEET during the germination process demonstrated a high sensitivity to this repellent. Plant cultures treated with a 0.01% concentration of DEET were characterized by a 40% inhibition of seed germination compared to the control group on day 10 [54].

Additionally, our studies revealed the additive toxic effects of the mixture of OMPs on *W. arrhiza* growth, indicating a significant interaction between them. For example, previous studies indicated that the mixture of both benzophenone-3 and BPH compounds inhibited the growth of the green alga *C. reinhardtii* more effectively than benzophenones used alone [51]. Another study indicated that different crop plant species (e.g., *Cucurbita moschata*, *Helianthus annuus*, *Ipomoea aquatica*, and *Zea mays*) showed various growth responses to the treatment with two organochlorine pesticides, lindane and α-END. *H. annuus* was the most tolerant to lindane, while *Z. mays* was the most resistant to the presence of α-END in the soil. These two pesticides applied together decreased the length of the root of *Z. mays* but did not affect the dry weights of the shoots and roots [55]. Thus, the mechanism of the joint toxicity of the used micropollutants may depend on the tested organisms.

Based on the obtained results and previously conducted studies, it can be concluded that the influence of contact with organic pollutants on the growth dynamics of floating macrophytes strongly depends on the type of the compound tested. When examining the effect of a mixture of four low-molecule benzotriazoles (at a total concentration of 0.4 mg/L) on *W. arrhiza*, a greater increase in plant weight was recorded compared to the control [29]. Similar observations were made when the same mixture was added to media in which floating plants of the common duckmeat *Spirodela polyrhiza* and mosquito fern (*Azolla caroliniana*) species were grown [56]. Despite the more or less intense negative impact of the tested OMPs on the plant growth observed in the conducted experiments, previous studies have shown the high effectiveness of *W. arrhiza* in removing DEET, TRC, and BPA [30]. From culture media enriched with the tested micropollutants at a concentration of 0.1 mg/L during 7-day contact with *W. arrhiza*, 88% of DEET and 98% of BPA and TRC were removed. The removal efficiency of BPA and TRC at a concentration of 0.5 mg/L was slightly lower but still exceeded 90%. The mechanism of removing pollutants from water by hydrophytes, in addition to the life processes of the plant and the microorganisms coexisting with it, also includes abiotic processes such as hydrolysis, photodegradation, and sorption. In the case of DEET, BPA, and TRC removal by *W. arrhiza*, the contribution of the plant uptake of the observed effect was estimated to range from 19 to 68% [30]. Organic pollutants taken into the plant are stored in cells in an unchanged form and/or undergo enzymatic modification (mainly degradation) or conjugation with glucose or glutathione [57]. Degradation can be intracellular or extracellular, and leads to complete mineralization or the formation of stable organic metabolites. It cannot be ruled out that the degradation products will be more toxic than the initial contaminant, but the vast majority of them will be stored in the plant and not in the purified water [57].

The effectiveness of phytoremediation depends on the plant’s welfare, which is closely related to the conditions in which it lives. One of the most important conditions affecting aquatic plants is the pH of their living environment. Plants of the Lemnaceae family can survive in a pH range of 3.5 to 10, but their optimal development occurs when the pH is close to 7 (6.5 to 7.5). A pH level that is too high or too low reduces the dynamics of plant growth and reproduction, and may cause problems with osmoregulation [58]. The light exposure time is important for the redox state of the plant and the response to biotic and abiotic stress. Increasing the daily light duration usually increases the efficiency of plant contaminant removal, but only to an optimal level. Excessive exposure to light causes abiotic stress, which reduces the metabolism and, therefore, OMP removal. Previous studies have shown that, for *W. arrhiza*, the optimal daily light exposure time is between 13 and 16 h. Since sorption on the surface of plants and their subsequent uptake are the important mechanisms responsible for phytoremediation, plant density is a relevant parameter of this process. The optimal removal of pollutants by *W. arrhiza* occurs when the mass of the plant equals 5 to 20 g of plant per liter of purified water [27,28,29,30]. A higher density is unfavorable, as it disturbs the biomass growth, morphology, and reproduction.

### 2.2. Proteins

The soluble protein level may be regarded as an indicator of both oxidative stress and as a growth parameter in plants. Our results confirmed the high toxicity of the mixture of all of the applied OMPs in the *W. arrhiza* cultures, because the protein level was decreased by 77.9% in comparison with the control (Figure 1). Among all of the organic compounds used in this experiment, DEET inhibited protein accumulation in *W. arrhiza* plants the most effectively (by 70.8–73.6%). Although the repellency and toxicological effects of DEET on insect viability and human health are well documented, there have been no attempts to study its influence on plant physiology and biochemistry, including the metabolite level. Our studies showed that DEET can be treated as a substance with potential herbicidal properties, which can act like a harmful contact pesticide, leading to protein degradation. A 47.4–62.3% decrease in the protein level was observed in *W. arrhiza* plants exposed to TRC. Similarly to our results, a significant decrease in the soluble protein concentration was also noted in both of the freshwater plants *E. crassipes* and *H. verticillata* exposed to TRC at concentrations from 0.05 to 0.5 mg/kg [49]. Wheat (*Triticum aestivum* L.) seedlings stressed by TRC have been also characterized by a lower protein content in comparison with the control [59]. This compound also showed long-term effects on the level of cellular biochemical components, such as lipids, proteins, and nucleic acids, in freshwater algae, e.g., *Asterococcus superbus*, *Chlorococcum* sp., *C. reinhardtii*, and *Eremosphaera viridis* [60]. TRC is an important stress factor leading to specific protein damage [61] and/or to the formation of reactive oxygen species (ROS), which can further contribute to the oxidative disruption of proteins [60].

A lower toxic effect on the protein concentration was observed in *W. arrhiza* cultures treated with BPA, because their level was reduced by 44.6–57.8% in relation to the control. Our observations agree with previous studies on soybean (*Glycine max*) plants, indicating that BPA reduced the activities of enzymes (e.g., nitrate reductase and nitrite reductase), which are critical for the production of protein in the roots of the seedlings of crop plants [62]. Similar results were also found in studies showing that duckweed (*Lemna minor*), a floating freshwater plant, in response to the highest concentration of BPA (50 mg/L), was characterized by lower contents of soluble proteins. In contrast, BPA, at lower concentrations of 1, 5, and 20 mg/L, did not exert a statistically significant effect on this parameter [63]. Comparative proteomics analysis performed on *Arabidopsis thaliana* plants indicated that BPA possesses high biological activity, influencing the synthesis of proteins responsible for the signaling, hormonal, and metabolic pathways playing a key role in the stability of plant growth and adaptation to stress [64].

The exogenous application of BPH decreased the protein content in *W. arrhiza* by 7.8–66.3% compared to the control plants. Thus, the toxicity of this organic micropollutant depends strongly on the concentration used in the experiment. The effect of this group of pollutants on the protein metabolism was confirmed in *Brassica rapa* under exposure to oxybenzone (benzophenone-3) [65]. Proteomics and metabolomics analyses showed that benzophenone-3 can induce ROS production, the peroxidation of membrane lipids, disturbances in root respiratory homeostasis, changes in the proteins involved in disease resistance interactions, carbon distribution, and nitrogen assimilation. Moreover, the accumulation of free amino acids due to protein degradation was observed in plants stressed with benzophenone-3 [65]. Endosulfans (α and β) were characterized by the lowest inhibitory effect on the soluble proteins in *W. arrhiza*, leading to a 1.5–37.3% decrease in their levels. In contrast to our results, the content of total soluble proteins increased in soybeans treated with endosulfan [66], indicating different patterns of plant responses to this micropollutant.

An increase in the content of soluble proteins was also recorded when *W. arrhiza* was exposed to a mixture of low-molecule benzotriazoles (BTRs), but was at the same level as in the control when this plant was exposed to municipal sewage containing phthalates [27,29].

### 2.3. Monosaccharides

In plants, sugars produced during photosynthesis play an essential role as vital sources of energy and carbon skeletons for organic compounds and storage components. Generally, OMPs used at lower concentrations (0.1 mg/L) stimulated the monosaccharide content in *W. arrhiza* plants (Figure 1). The highest increase, by 59.0% and 58.8%, in the content of this biochemical parameter was observed in *W. arrhiza* fronds treated with 0.1 and 1 mg/L α-END, respectively. The lower rise in the monosaccharide level was reported for DEET (9.2–53.8%), β-END (10.6–38.8%), and BPH (4.4–11.0%). On the other hand, BPA and TRC at a low concentration of 0.1 mg/L were characterized by stimulatory influence on monosaccharide accumulation, whereas, at a high concentration (1 mg/L), they did not exert or inhibit the content of this biochemical component. The content of monosaccharides has not been changed under the influence of the mixture of all of the studied organic compounds.

The knowledge concerning the effects of these micropollutants in animals is rather well documented. However, data remain insufficient in plants, especially regarding their impact on sugar accumulation and metabolism. For example, the BPA treatment positively affected the production of soluble sugars in the roots of *Vicia faba* [67]. On the other hand, another study showed a drastic reduction in the sugar content in three species of cyanobacteria, *Anabaena fertilissima*, *Aulosira fertilissima*, and *Westiellopsis prolifica*, growing in the presence of endosulfan [68]. The increase in the content of monosaccharides may be connected with their role as signaling molecules in plant defense responses to various abiotic stress factors. For example, a marked enhancement in disaccharide (sucrose) and monosaccharide (glucose and fructose) concentrations has been reported in plants under saline, drought, and cold stress. These soluble sugars are responsible for the synthesis of organic compounds, carbon storage, the stabilization of cellular membranes, osmoprotection, the protection of proteins during stress conditions, and antioxidants involved in the scavenging of ROS in plant cells [69]. Thus, the accumulation of sugars in response to the studied micropollutants may provide better tolerance of *W. arrhiza* plants to OMPs. The stimulating effect on the carbohydrate content in *W. arrhiza* tissues was described in works concerning the removal of a mixture of BTRs and municipal wastewater by phytoremediation using this plant [27,29].

### 2.4. Chlorophylls

**Figure 2:**
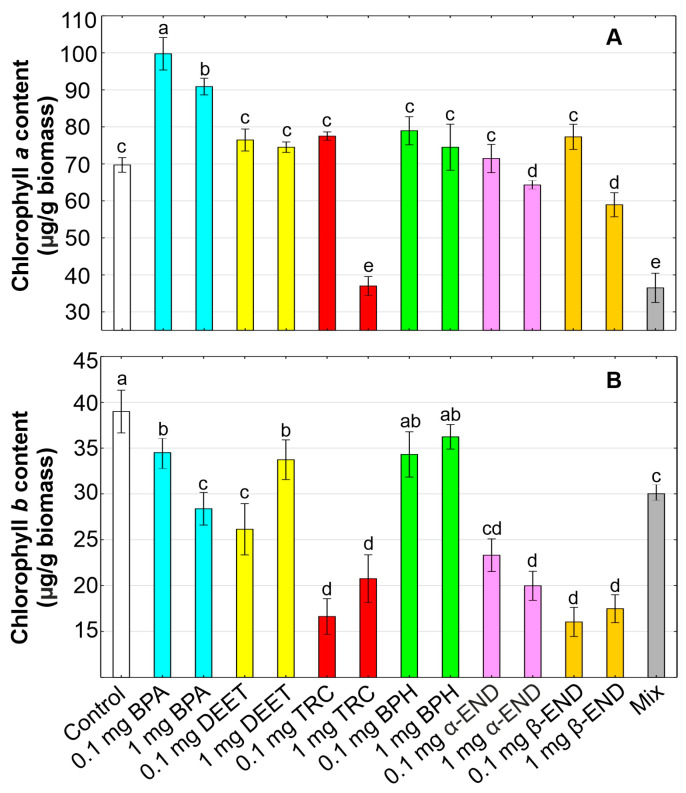
The content of chlorophyll *a* (**A**) and *b* (**B**) in *Wolffia arrhiza* cultures under the influence of the following micropollutants: BPA (0.1 and 1 mg/L), DEET (0.1 and 1 mg/L), TRC (0.1 and 1 mg/L), BPH (0.1 and 1 mg/L), α-END (0.1 and 1 mg/L), and β-END (0.1 and 1 mg/L), as well as their mixture, in relation to the control on the 7th day of culture. Data are the means of three independent experiments ± SD. Treatments with at least one letter being the same indicate a non-significant difference according to Tukey’s post hoc test.

The changes in the content of chlorophyll *a* and *b* are important parameters for assessing photosynthetic activity and can be treated as markers of plant viability under the various conditions of the environment. Our results indicated that the content of chlorophyll *a* decreased by 7.7% and 15.4% in *W. arrhiza* subjected to α-END and β-END at a concentration of 1 mg/L, respectively (Figure 2). Both of the endosulfan forms at a lower concentration 0.1 mg/L did not induce a statistically significant effect on the level of this photosynthetic pigment. Additionally, chlorophyll *b*’s content was the most inhibited by the presence of α-END and β-END by 40.2–58.9% in relation to the control. Our results are confirmed by previous studies indicating that the accumulation of photosynthetic pigments (chlorophyll *a*, carotenoids, and phycocyanin) in cyanobacterium *Plectonema boryanum* was inhibited by the endosulfan treatment, and this decrease was found to be dose-dependent [70]. Endosulfan also inhibited the relative growth rate, chlorophyll content, and photosynthetic O_2_ evolution in the aquatic plant *Azolla microphylla* [71].

The decrease in the chlorophyll *a* and *b* content by 46.9% and 46.8%, respectively, has been also noted in *W. arrhiza* treated with 1 mg/L of TRC. On the other hand, the lower toxicity of this organic contaminant at a concentration of 0.1 mg/L on the chlorophyll level was observed in the studied plant. Similarly, the results obtained on the aquatic plants *E. crassipes* and *H. verticillata* indicated that the chlorophyll concentrations in the leaves of these two plant species increased in response to TRC used at low concentrations and decreased in plants growing in the presence of higher concentrations of TRC in sediments [49]. The reduction in the level of chlorophylls under the influence of endosulfan and TRC might be owing to several reasons, such as the inhibition of their biosynthesis and/or oxidative degradation. The disturbance in the photosynthetic apparatus caused by these micropollutants can potentially disrupt the metabolic process of the aquatic plant *W. arrhiza*, leading to growth inhibition.

On the other hand, less toxicity on photosynthetic pigments in *W. arrhiza* was observed in the case of other organic compounds, such as BPA, DEET, and BPH. The increase in the level of chlorophyll *a* by 30.4–43.1%, 6.9–13.3%, and 6.9–9.7% was reported in cultures treated by BPA, BPH, and DEET, respectively. However, there was a decrease in chlorophyll *b* by 11.5–27.2%, 7.1–12.0%, and 13.5–32.9% in the plants treated with BPA, BPH, and DEET, respectively. Thus, the results indicated an increased chlorophyll *a*/*b* ratio in *W. arrhiza* plants treated with these three OMPs. Adjusting the chlorophyll *a*/*b* ratio may be an integral feature of plant acclimation to stressful conditions of the environment. The literature data indicate that the chlorophyll *a* and carotenoid content was significantly elevated in 4-week-old plants of *A. thaliana* exposed to 5 mg/L of BPA [72]. Additionally, higher activities of key enzymes involved in chlorophyll synthesis at different growth stages, resulting in increases in the chlorophyll levels and the net photosynthetic rate, were observed in soybean (*G. max*) exposed to BPA used at a low dose (1.5 mg/L) [73]. This finding, however, was different from a previous study showing that the green alga *Chlamydomonas reinhardtii* and cyanobacterium *Microcystis aeruginosa* treated with BPH were characterized by lower contents of chlorophylls in cells [74]. The results indicate the potentially harmful effects of benzophenones on the process of photosynthesis and that the mechanism of action can vary with the species. Moreover, some of the results are in accordance with our studies, indicating the positive effect of DEET on chlorophyll levels. For example, other pesticides, such as aldicarb, carbofuran, fenamiphos, fensulfothion, and phorate, used at low concentrations improved the plant growth (expressed as the plant length as well as the weight), pod numbers, root nodulations, and chlorophyll content in the chickpea (*Cicer arietinum*) plant [75].

The exposure of *W. arrhiza* to the mixture of organic compounds reduced chlorophyll *a* by 47.6% and chlorophyll *b* by 23.0% in relation to the control culture, leading to growth inhibition. The toxicity of the combined organic compounds to the chlorophyll level is stronger than that of single micropollutants used alone. Thus, the combined toxicity of all of the tested OMPs to *W. arrhiza* was evaluated as a synergistic effect on the chlorophyll content. Similarly, a significant reduction in the chlorophyll content in *W. arrhiza* tissues, reaching up to 80%, was observed under the influence of a mixture of benzotriazoles as well as municipal wastewater [27,29]. A decrease in the concentration of chlorophyll *a* and chlorophyll *b* also occurred under the action of BTRs on other small floating macrophytes, with *A. caroliniana* being characterized by a significantly higher sensitivity in comparison to *S. polyrhiza* [56].

### 2.5. Carotenoids

Carotenoids, which are divided into carotenes and xanthophylls, are also photosynthetic pigments that have been studied in detail in response to BPA, DEET, TRC, BPH, α-END, and β-END. Carotenoids are essential pigments, along with chlorophylls, involved in photosynthesis, also playing a key role as photo-protectors, antioxidants, color attractants, and precursors of plant hormones [76].

**Figure 3:**
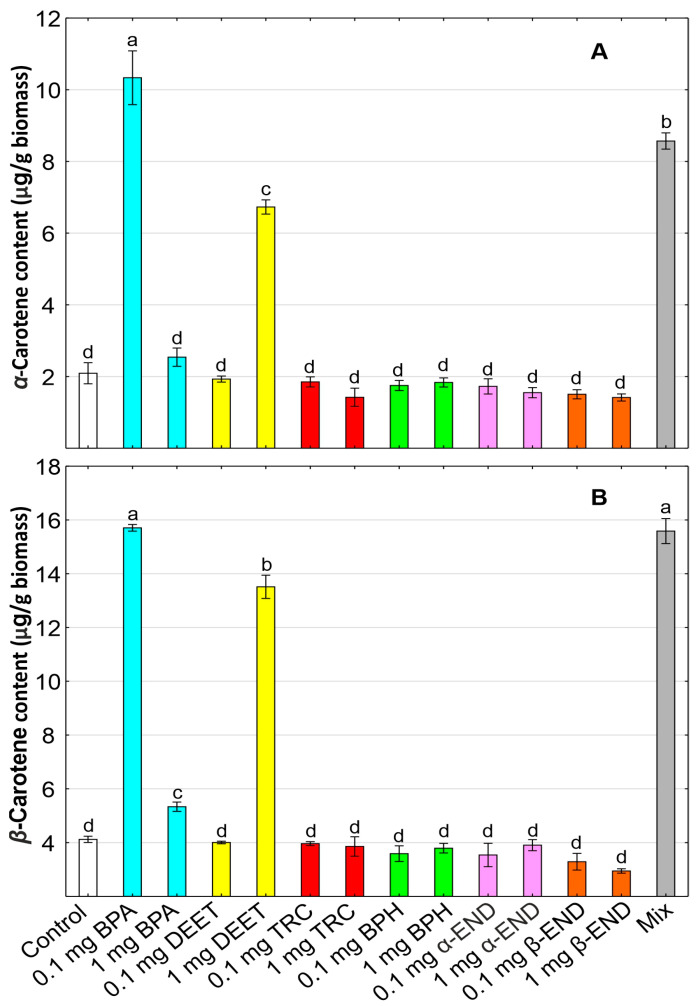
The content of α-carotene (**A**) and β-carotene (**B**) in *Wolffia arrhiza* cultures under the influence of the following micropollutants: BPA (0.1 and 1 mg/L), DEET (0.1 and 1 mg/L), TRC (0.1 and 1 mg/L), BPH (0.1 and 1 mg/L), α-END (0.1 and 1 mg/L), and β-END (0.1 and 1 mg/L), as well as their mixture, in relation to the control on the 7th day of culture. Data are the means of three independent experiments ± SD. Treatments with at least one letter being the same indicate a non-significant difference according to Tukey’s post hoc test.

Among carotenoids, α-carotene and β-carotene were increased in BPA-treated plants by 21.4–394.0% and 29.5–281.2%, respectively, in comparison with the control (Figure 3). Similar to our results, the carotenoid content was elevated in 4-week-old *A. thaliana* growing in the presence of BPA at low concentrations [72]. The increase in the level of carotenes in *W. arrhiza* in response to BPA may be a part of the adaptative strategy and biochemical tolerance to OMPs, as it has been confirmed in the green alga *Acutodesmus obliquus* growing in the presence of oxidized BPAs [77]. The increase in the level of α-carotene and β-carotene by 221.7% and 227.9%, respectively, was noted in *W. arrhiza* in response to DEET. Similar to our results, the carotenoid content was increased in peach (*Prunus persica*) leaves treated with other pesticides, e.g., acetamiprid, chlorantraniliprole, chlorpyrifos, cyantraniliprole, deltamethrin, indoxacarb, pyriproxyfen, and spinetoram [78], and in lettuce (*Lactuca sativa*) leaves growing in the presence of the following pesticides: benomyl, chlorpropham, iprodione, propyzamide, and vinclozolin [79].

On the other hand, the presence of TRC, BPH, α-END, and β-END in the growth medium reduced the level of both carotenes in *W. arrhiza* plants. A severe depletion in carotenoids has been also observed in the diatom *Phaeodactylum tricornutum* treated with TRC at high concentrations [80]. Other studies revealed that benzophenone can induce the structural and UV photon energy input (on which it is highly dependent) destruction of β-carotene [81]. The decrease in the carotenoid level was also reported in *P. leptostachyus* plants exposed to organochlorine pesticide endosulfan [39]. The changes in the concentration of carotenes provide information about the toxicity of OMPs in plants and their ability to endure abiotic stress. Therefore, due to the significant decrease in the content of carotenes in response to exogenously applied TRC, BPH, and α-END, as well as β-END, it is evident that these pollutants may lead to the inhibition of plant growth, photosynthesis, and viability. A similar trend was observed in the case of xanthophylls (astaxanthin, neoxanthin, cryptoxanthin, lutein, violaxanthin, and zeaxanthin) in *W. arrhiza* cultures treated with various OMPs (Table 2).

The content of xanthophylls was stimulated in response to BPA and DEET. In contrast, TRC, BPH, and α-END, as well as β-END, had inhibitory effects on the content of these pigments. Xanthophylls are involved in counteracting photoinhibition and free radical quenching during photosynthesis. These pigments protect the photosynthetic apparatus against oxidative stress generated by chemical toxic substances [76]. Therefore, the increase in the xanthophyll content in *W. arrhiza* plants treated with BPA and DEET may be a part of the adaptative strategy against abiotic stress generated by these two toxins. On the other hand, the decrease in the xanthophyll content under the influence of TRC, BPH, and α-END, as well as β-END, may lead to the disruption in the process of photosynthesis and the inhibition of *W. arrhiza* growth. Reduced carotenoid levels also affect the oxidative stress, as several carotenoids can counteract lipid peroxidation and scavenge ROS. Similarly to our results, the level of pigments involved in the xanthophyll cycle (diadinoxanthin and diatoxanthin) showed a significant reduction in the diatom *P. tricornutum* in response to high concentrations (50 and 100 μg/L) of TRC [80]. Furthermore, the decrease in the xanthophyll level has been also noted in *W. arrhiza* plants exposed to the mixture of all of the studied OMPs, indicating the synergistic toxic effect of all of the used chemical substances on the photosynthetic apparatus. Additionally, the degradation of photosynthetic pigments is a typical plant response to abiotic stress factors such as chemical pollutants. These findings show that the mixture of all of the OMPs is extremely toxic to *W. arrhiza* cultures, and may induce changes in the content of photosynthetic pigments and their composition, bringing to light that the coexistence of various micropollutants poses risks to the functioning of aquatic environments. A similar effect on the content of carotenes and xanthophylls in *W. arrhiza* tissues was observed under the influence of the BTR mixture [29]. An increase in the content of carotenoids was registered when the plant was exposed to municipal sewage, which may indicate the activation of a strategy aimed at counteracting the toxic effects caused by oxidative stress [27]. In studies on other small floating macrophytes, it was noted that changes in the carotenoid content under the influence of organic pollutants strongly depended not only on the type of chemical compounds but also on the tested plant species. While there was a significant decrease in the concentration of these pigments in the tissues of *A. caroliniana*, *S. polyrhiza* demonstrated the ability to increase the production and accumulation of carotenoids in its body [56].

### 2.6. Oxidative Stress Markers

**Figure 4:**
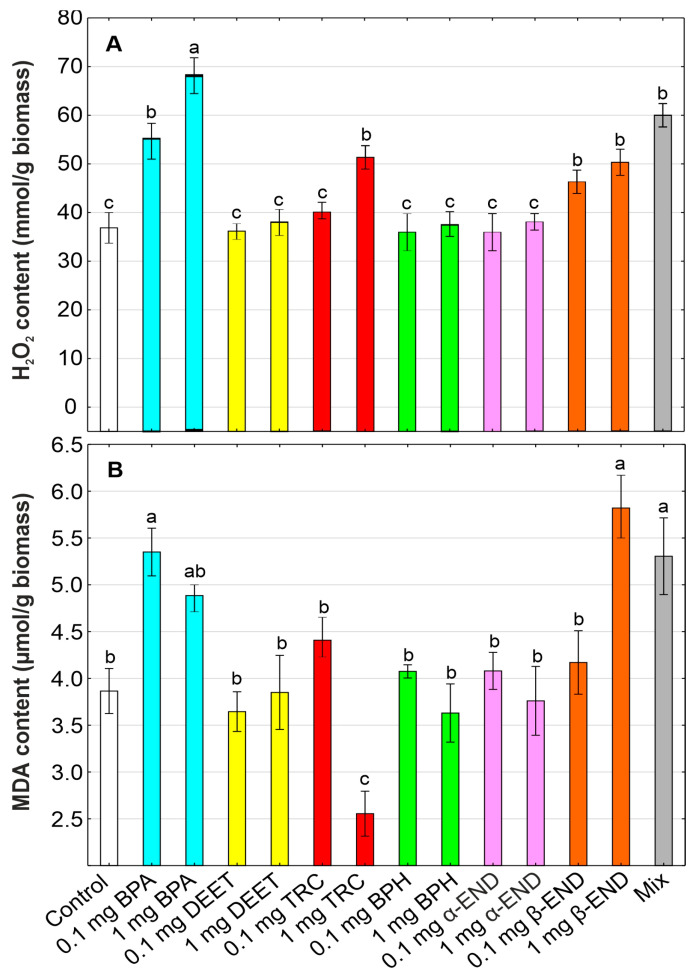
Malondialdehyde (MDA) (**A**) and hydrogen peroxide (H_2_O_2_) (**B**) contents in *Wolffia arrhiza* cultures under the influence of the following micropollutants: BPA (0.1 and 1 mg/L), DEET (0.1 and 1 mg/L), TRC (0.1 and 1 mg/L), BPH (0.1 and 1 mg/L), α-END (0.1 and 1 mg/L), and β-END (0.1 and 1 mg/L), as well as their mixture, in relation to the control on the 7th day of culture. Data are the means of three independent experiments ± SD. Treatments with at least one letter being the same indicate a non-significant difference according to Tukey’s post hoc test.

Malondialdehyde (MDA) and hydrogen peroxide (H_2_O_2_) contents were determined to evaluate the degree of oxidative stress affecting *W. arrhiza* plants after the influence of different organic toxicants alone and their mixture (Figure 4). MDA provides an analytical index to indicate the lipid peroxidation of cellular membranes. The larger the content of MDA, the less the biochemical stability of the membranes is observed. The highest increase by 52.0% in the MDA level was observed in the cultures treated with β-END at a concentration of 0.1 mg/L in relation to the control. The presence of BPA in the medium also induced a significant rise (by 26.4–38.4%) in the MDA content in comparison with the control culture. The TRC treatment showed a somewhat different pattern, where a significant decrease in the MDA level by 33.9% was observed at a low concentration of 0.1 mg/L of TRC, whereas this parameter was stimulated by 14.0% in response to higher concentrations of TRC (1 mg/L). Generally, OMPs used at low doses were characterized by lower stimulatory effects on lipid peroxidation. In contrast, they generated extensive oxidative stress at high doses, showing that ROS severely damaged the cell membrane. Thus, we can speculate that low concentrations of exogenous organic compounds (0.1 mg/L) could improve the tolerance of *W. arrhiza* to toxins present in aquatic ecosystems, leading to its better growth and development. The mixture of all of the organic compounds enhanced the lipid peroxidation, indicating the synergistic effect of the used OMPs on the oxidative cellular status of *W. arrhiza*.

In summary, MDA accumulations were the highest in *W. arrhiza* grown in β-END-, BPA-, and TRC-contaminated media. This observation agrees with previous results, indicating the presence of endosulfan-induced lipid peroxidation in Bayo beans (*Phaseolus leptostachyus*) [39]. Similarly, plants, e.g., *Arundo donax*, *Phragmites australis*, and *Typha latifolia*, treated with BPA were characterized by a higher level of oxidative stress, as evidenced by the higher MDA production [82]. Additionally, a significant increase in the MDA content in the green alga *Chlorella vulgaris* confirmed that the cell membranes were severely damaged by the ROS generated in response to 1.05 mg/L of TRC [83,84]. Our study indicated that lipid peroxidation may be treated as a common phenomenon in plants under various stress factors, and MDA may be used as an important indicator of the physiological status of plant growth under the influence of hazardous organic compounds. BPH, DEET, and α-END did not significantly affect lipid peroxidation in *W. arrhiza* biomass. In contrast to our results, BPH and benzophenone-3 slightly increased the MDA content in the cyanobacterium *M. aeruginosa*, causing moderate oxidative stress in the cells [85]. Similarly, Jin et al. [86] studied the physiological responses of oilseed rape (*Brassica napus*) to stress by the herbicide ZJ0273 (propyl 4-(2-(4, 6-demethoxy pyrimidin-2-yloxy) benzylamino) benzoate), and the results indicated that the MDA contents showed a positive correlation with the increasing dose of ZJ0273. Moreover, ZJ0273 applied at a low concentration did not induce lipid peroxidation in *B. napus* [86].

Under abiotic stress, plants may increase the production of ROS, including hydrogen peroxide (H_2_O_2_). The dismutation of the superoxide radical forms H_2_O_2_, which is a moderately reactive molecule often used as a marker of oxidative stress. H_2_O_2_ may also be treated as an essential secondary signaling messenger involved in various growth and developmental processes, stress signaling pathways, and biochemical reactions. However, when this compound is produced in excess, it becomes toxic and harmful to the cell metabolism, even causing cell death [63,70,82,85].

Similar to the MDA level, the pattern of the accumulation of H_2_O_2_ in *W. arrhiza* plants treated with BPA (0.1 mg/L and 1 mg/L), DEET (0.1 mg/L and 1 mg/L), TRC (0.1 mg/L and 1 mg/L), BPH (0.1 mg/L and 1 mg/L), α-END (0.1 mg/L and 1 mg/L), and β-END (0.1 mg/L and 1 mg/L) was characterized by the same trend. The highest increase in the H_2_O_2_ level by 49.5–85.3% was observed in the plants treated with BPA. Plants subjected to β-END stress were also characterized by higher levels of H_2_O_2_ (by 24.8–35.3%) compared to the control. Additionally, TRC was also able to induce the formation of ROS, which can further contribute to the oxidative damage of plant cells, because the H_2_O_2_ content was stimulated by 9.4–43.0% in relation to the control. The level of oxidative stress was the highest in the *W. arrhiza* plants treated with BPA, β-END, and TRC at higher concentrations (1 mg/L). These three compounds’ toxic effect was slighter when applied at low concentrations (0.1 mg/L). H_2_O_2_ concentrations, on the other hand, did not vary significantly after the treatments with DEET, BPH, and α-END. Thus, we can speculate that the *W. arrhiza* plants did not show any symptoms of oxidative stress because the H_2_O_2_ level was at the control level. Additionally, the mixture of all of the studied xenobiotics generated oxidative stress, as evidenced by a higher (increase of 61.6%) accumulation of ROS than the control culture.

The obtained results confirmed the previous studies showing the increased content of H_2_O_2_ in the leaves of the model plant *A. thaliana* in the presence of BPA [87], in the wheat *Triticum aestivum* roots exposed to TRC [88], and in *Phaseolus leptostachyus* grown in endosulfan-contaminated soil [39]. These results are also similar to those reported by An et al. [59], suggesting that oxidative stress may be a common biochemical symptom observed in plants in response to TRC application. It has been suggested that H_2_O_2_ accumulation contributes to restricted cell expansion, enhanced cell wall rigidification, the increased production of lignins, and the inhibited biosynthesis of auxins (auxins are phytohormones responsible for normal plant growth), leading to the ultimate inhibition of plant growth and development [88]. The generation of ROS induced by BPA, TRC, and β-END may also be treated as the start of the cascades of cellular signaling molecules involved in the pathways responsible for the degradation of various biochemical components, such as carbohydrates, lipids, proteins, and DNA. For that reason, ROS accumulation may contribute significantly to the phytotoxic potential of these three organic OMPs in *W. arrhiza* plants. Enhanced ROS generation may also trigger the defense mechanisms in plant cells to cope with the oxidative stress damage, which is important in signaling pathways and plant growth regulation [87].

Correspondingly to the results of the test performed on *W. arrhiza*, a short exposure to DEET caused no effects on the lipid peroxidation and H_2_O_2_ levels in the aquatic midge *Chironomus riparius*, despite the significant inhibition of the antioxidant enzyme (e.g., catalase and glutathione-S-transferase) activities and total glutathione contents [89]. Other results showed that plants treated with benzophenone-3 could reconfigure the mitochondrial electron transport chain to bypass oxidative damage components, remove excessively accumulated ROS, enhance the efficiency of the antioxidant system, and detoxify the harmful lipid peroxides present in membranes [65]. So, we can speculate that a low H_2_O_2_ content under BPH stress may result from the higher activity of the antioxidant system in response to this compound; however, enzymatic and non-enzymatic antioxidants were not studied in *W. arrhiza*. Additionally, other studies revealed that BPH and its chemical analogs displayed an important antioxidant activity and low cytotoxicity, and could decrease the reactive oxygen species production in mammal cells [90]. Based on the obtained results, we can suggest that BPH may act as an effective scavenger of H_2_O_2_ in *W. arrhiza*. Furthermore, a moderate effect of DEET, BPH, and α-END on the oxidative status of the plant cell in our experimental system was observed.

Generally, H_2_O_2_ production and lipid peroxidation patterns were similar in *W. arrhiza* cultures treated with various organic pollutants, indicating that BPA, TRC, and β-END can generate oxidative damage. In contrast, DEET, BPH, and α-END did not induce a statistically significant impact on the oxidative stress markers. Other studies conducted with *W. arrhiza* as a test organism have shown that, in most cases, the H_2_O_2_ and MDA contents increase in response to contact with organic pollutants [29].

## 3. Materials and Methods

### 3.1. Chemicals and Materials

Analytical BPA, DEET, TRC, BPH, α-END, and β-END standards (with a purity of at least 97%) were obtained from Merck, Darmstadt, Germany. The 1 mg/mL stock solutions were prepared by dissolving 10 mg of each analytical standard in 10 mL of ethanol (for the liquid chromatography grade; Merck, Darmstadt, Germany). All of the reagents used to prepare the culture media were purchased from Chempur, Piekary Śląskie, Poland, and Avantor Performance Materials, Gliwice, Poland.

### 3.2. Plant Material and Growth Conditions

*Wolffia arrhiza* (L.) Horkel ex Wimm. was cultivated for 7 days at 22 ± 0.5 °C in a 12:12 (light/dark) cycle. Plant cultures were illuminated from above with a light intensity of 50 µmol/m^2^/s at the surface of the growing containers. Plants were cultivated in glass crystallizers with a capacity of 2 L and a diameter of 20 cm, including 1 L of Hunter’s medium, covered with a polyethylene foil, and completely penetrable for light [29,31]. The following reagents were used to prepare the sterile mineral Hutner’s medium [91]: Na_2_EDTA, MgSO_4_·7H_2_O, NH_4_VO_3_, FeSO_4_·7H_2_O, MnCl_2_·4H_2_O, Co(NO_3_)_2_·6H_2_O, KH_2_PO_4_, KNO_3_, Ca(NO_3_)_2_·4H_2_O, KOH, ZnSO_4_·7H_2_O, Na_2_MoO_4_·2H_2_O, H_3_BO_3_, and CuSO_4_·5H_2_O. Then, the medium was diluted with water in a ratio of 1:50. The following chemical xenobiotics belonging to the OMPs, BPA, DEET, TRC, BPH, and two endosulfan isomers, α-END and β-END, as well as their mixture, were added to the growth medium to obtain the concentrations of 0.1 and 1 mg/L. The changes in the plant growth were expressed as dry mass. The contents of cellular components and oxidative stress markers in response to various micropollutants were determined on the 7th day of the *W. arrhiza* cultivation. The obtained results were compared to the control.

### 3.3. Plant Growth Determination

For the dry mass determination, the plant cultures were filtered, washed three times with distilled water, kept on filter paper for a few minutes to remove the excess liquid, and dried at 60 °C for 24 h. Then, the dried plants were weighed.

### 3.4. Determination of Monosaccharide and Water-Soluble Protein Contents

For the monosaccharide determination in *W. arrhiza*, the cultures were first collected by filtration, and then the pellets (0.1 g) were extracted with ethanol. The arsenomolybdate [92] method, also using cuprum reagent, was used to measure the content of reducing sugars (monosaccharides) in the plant tissue.

The measurement of the soluble protein content was started from the homogenization of the biomass. Then, the homogenate was centrifuged for 10 min at 12,000× *g,* and an aliquot of the extract was used to determine the protein content following the Bradford method [93]. Coomassie Brilliant Blue G-250, which can bind to protein rapidly, and the complex remains dispersed in the solution for a long time were applied for the spectrophotometrical protein analysis. Bovine serum albumin was used as the analytical standard of the soluble proteins.

### 3.5. Determination of Photosynthetic Pigments

The homogenization of the *W. arrhiza* for the determination of the contents of chlorophyll *a* and *b*, as well as individual carotenoids (carotenes and xanthophylls) was performed by placing fresh plants in methanol (99.9%) in a bead mill (50 Hz and 10 min, TissueLyser LT; Qiagen GmbH, Düsseldorf, Germany) during 3 min for the tissue disruption and pigment extraction [94]. The extracts were filtered using 0.45 μm PTFE filters (A&A Biotechnology, Gdansk, Poland) for their purification. Clean extracts of photosynthetic pigments were analyzed using an Agilent 1260 Infinity Series High-Performance Liquid Chromatography (HPLC) system (Agilent Technologies, Inc., Santa Clara, CA, USA). The HPLC system contained a refrigerated autoinjector with a 500 μL sample loop for large-volume injections, a thermostatted column oven, a quaternary pump with an in-line vacuum degasser, and a photo-diode array detector. The HPLC column Eclipse XDB C_8_ (150 mm × 4.6 mm) (Agilent Technologies, Inc., Santa Clara, CA, USA) was used for the pigment separation and analysis at 25 °C. The visible absorbance spectra of the plant pigments from 350 to 750 nm were monitored using a photo-diode array detector with 20 nm bandwidths. The injection volume for the photosynthetic pigment extracts was 200 µL. Mobile phase A consisted of a mixture of methanol, acetonitrile, and 0.25 M of aqueous pyridine solution (pH of 5.0) in a proportion of 50/25/25 (*v*/*v*/*v*), whereas mobile phase B was a mixture of methanol, acetonitrile, and acetone in a proportion of 20/60/20 (*v*/*v*/*v*). The gradient was linear, starting from 100% of eluent A and returning to 100% at the 40th min of the experiment [95]. Flow rates were adjusted to maintain backpressure below 180 bar (1 mL/min). The analytical data were integrated using Agilent OpenLAB ChemStation Edition C.01.09 for LC systems (Agilent Technologies, Inc., Santa Clara, CA, USA).

### 3.6. Malondialdehyde and Hydrogen Peroxide Determination

Lipid peroxidation was determined by measuring the amount of total malondialdehyde (MDA) in the plant tissue according to the method in [96]. Plant tissue (0.1 g) was homogenized in 50 mM phosphate buffer (pH of 7.0) in a bead mill for 3 min. Then, homogenate was centrifuged at 15,000× *g* for 15 min. The obtained supernatant was treated with 0.5% thiobarbituric acid (TBA) in 20% trichloroacetic acid (TCA), and the mixture was heated at 95 °C for 30 min in the water bath. Then, the reaction mixture was cooled in an ice bath. After centrifugation (10 min at 12,000× *g*), the absorbance of the supernatant was measured at 532 nm. The value for the nonspecific absorption of each sample at 600 nm was also recorded and subtracted from the absorbance recorded at 532 nm. The contents of MDA in the plant extracts were calculated using an extinction coefficient of 155 mM/cm.

The hydrogen peroxide (H_2_O_2_) level in *W. arrhiza* was measured spectrophotometrically after the reaction with KI using the method by Alexieva et al. [97]. The reaction mixture consisted of 0.1% TCA, plant tissue extract supernatant, 100 mM of K-phosphate buffer, and a reagent (a 1 M solution of KI *w*/*v* prepared in fresh distilled water). The blank probe consisted of 0.1% TCA without plant extract. The reaction mixture was kept in darkness for 1 h. The absorbances of the reaction mixtures were measured at a wavelength of 390 nm. The amount of this reactive oxygen species (ROS) was calculated using a standard curve prepared with known and fresh-prepared concentrations of H_2_O_2_. The contents of oxidative stress markers were calculated in 1 g of biomass of *W. arrhiza* plants.

### 3.7. Statistical Analysis

Each measurement was performed with three replicates, and each experiment was conducted at least twice at different times. Before selecting the appropriate statistical analysis method, the data were subjected to tests for normality (Shapiro–Wilk test) and homogeneity of variances (Levene’s test), and both the normality of the data and the homogeneity of the variances were confirmed. No significant outliers were identified within the data set. Consequently, the data were analyzed using an F-test and one-way ANOVA to identify the statistically significant differences between the calculated arithmetic means. Mean comparisons were conducted using Tukey’s post hoc test (TIBCO Software Statistica version 13.3). A significance level was set at *p* < 0.05 for all of the statistical tests and comparisons.

## 4. Conclusions

Micropollutants may be present in trace amounts among different environments and living organisms. Commonly known groups of micropollutants include personal care products (e.g., TRC and BPH), pesticides (e.g., DEET and endosulfans), and industrial chemicals (BPA), which might pose possible threats to ecological environments such as water, soil, and atmosphere. Although their effects on aquatic ecosystems are not very well known yet, there are strong suggestions, considering their acute and chronic impacts not only on animals but also on plant organisms. Information on this impact is important for understanding the plant physiology during stress conditions and the application of floating macrophytes in phytoremediation, which is a cheap and environmentally friendly method of removing micropollutants. Therefore, the influences of BPA, DEET, TRC, BPH, α-END, and β-END, as well as their mixture, were studied in *W. arrhiza* in detail, including on the plant growth, the contents of cellular components (photosynthetic pigments, proteins, and monosaccharides), and oxidative stress markers. The biological activity of all of the analyzed OMPs depends on their physical properties, chemical structure, and bioavailability in aquatic environments. All of the pollutants used in our experimental system were characterized by inhibitory effects on *W. arrhiza* growth (Table 3).

The chlorophyll *a* level was increased in response to BPA, DEET, and BPH, whereas a decrease in chlorophyll *a* was noted in *W. arrhiza* grown in the presence of TRC and both endosulfans. On the other hand, all of the studied xenobiotics reduced the chlorophyll *b* content. An increase in the accumulation of carotenoids was reported in the plants exposed to BPA and DEET. In contrast, their level was reduced in the cultures treated with TRC, BPH, and both endosulfans in relation to the control. The results indicate the various impact of OMPs on the function of the photosynthetic apparatus in *W. arrhiza* cells. All of the organic compounds stimulate the degradation of proteins. On the other hand, the level of monosaccharides was increased in response to xenobiotics. The disturbance in the membrane stability, expressed as higher MDA levels and a rise in the H_2_O_2_ concentrations, were observed in the plants exposed to BPA, TRC, and β-END, whereas DEET, BPH, and α-END had no statistically significant effect on the oxidative stress. The mixture of all of the organic pollutants may have the highest toxic effects on the plant growth and cellular components, as evidenced by the lower contents of proteins, sugar, and photosynthetic pigments, as well as a burst of ROS accumulation, bringing to light that the coexistence of various micropollutants poses risks to aquatic environments.

To explore the changes in the cellular functions caused by organic pollutants, we have summarized that organic chemicals may possess different impacts on plant growth and metabolism, and probably show different mechanisms of action in the aquatic plant *W. arrhiza*. This research has improved our knowledge about the biological activity of OMPs, which, despite their rather negative effect on plant growth, cannot be banned entirely because they are important for crop production, human health, and industry. An improved understanding of the effects of these organic chemicals on plants is also important from the point of view of planning the processes of their removal from water and wastewater by phytoremediation, and for searching for solutions to improve the efficiency of this process.

## Figures and Tables

**Table 1:** Basic information about the tested compounds.

Name	Structure	MW (g/mol)	CAS	pK_a_	logK_ow_	Application Effects on Organisms
BPA Bisphenol A	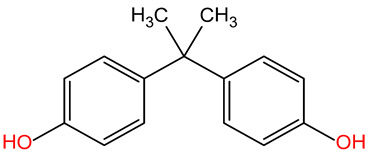	228.11	80-05-7	10.3	3.64	Used as a substrate for the production of polycarbonates and epoxy resins, and, therefore, many products such as food containers, the inner protective layers of cans, medical products, thermal paper, electronic devices, and dental fillings; it harms the hormonal balance of organisms; exposure is associated with adverse health effects, including diabetes, obesity, reproductive disorders, cardiovascular disease, breast cancer, and birth defects [34].
DEET *N*,*N*-Diethyl- *m*-toluamide	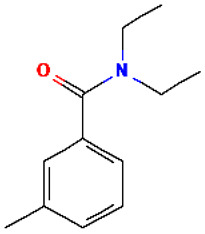	191.27	134-62-3	-	2.02	An insect repellent that is used in products (liquid sprays, lotions, and sticks) to prevent bites from insects, including ticks, flies, mosquitos, and some parasitic worms; toxic for some species of freshwater zooplankton and algae (significant biomass decline); have a slight toxicity for freshwater fish such as rainbow trout [35].
TRC Triclosan	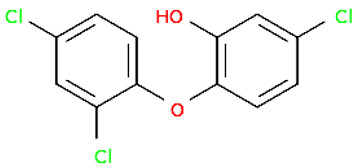	289.54	3380-34-5	7.9	4.76	Effective agent against bacteria, as well as some fungi and protozoa; widely used as an antiseptic, preservative, and disinfectant in healthcare, cosmetics, household cleaning products, the surface of medical devices, and kitchen utensils; bioaccumulates and poses a hazard to aquatic biota; can lead to a host of negative consequences in humans, including impaired thyroid function, endocrine disruption, developmental disorders, oxidative stress, and liver carcinogenesis [36].
BPH Benzophenone	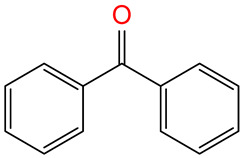	182.22	119-61-9	-	3.18	Used as a UV filter, and it is also a degradation product of oxybenzone (OXB), one of the most popular UV protection agents; BPH and OXB are used in the production of plastics and coatings, adhesives, insecticides, pharmaceuticals, and cosmetics; BPH disrupts the functioning of the endocrine system, and limits the reproductive and developmental abilities of organisms; suspected to have neurotoxic and carcinogenic effects [37].
α-END α-Endosulfan	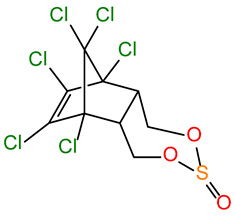	406.93	959-98-8	-	4.94	Since the 1950s, it has been used as an insecticide and acaricide on a wide variety of food crops, including tea, coffee, fruits and vegetables, rice, cereals, maize, sorghum, and also as a wood preservative; banned by the Stockholm Convention in 2011 due to its risk to human health and the environment; still used in some countries, mainly in Asia; toxic by inhalation, skin absorption, or ingestion; has significant acute toxicity, the ability to bioaccumulate, and disrupt the endocrine system of organisms; highly neurotoxic to both insects and mammals, including humans [38].
β-END β-Endosulfan	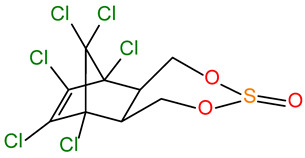	406.93	33213-65-9	-	4.32	

**Table 2:** Content of xanthophyll pigments in *W. arrhiza* biomass treated with different concentrations of micropollutants in relation to the control on the 7th day of cultivation. Data are the means of three independent experiments ± SD. Treatments with at least one letter being the same indicate a non-significant difference according to Tukey’s post hoc test.

Treatment	Xanthophyll Content (µg/g Biomass)						
	Antheraxanthin	Astaxanthin	Kryptoxanthin	Lutein	Neoxanthin	Violaxanthin	Zeaxanthin
Control	0.61 ± 0.13 ^d^	0.54 ± 0.05 ^c^	2.26 ± 0.22 ^b^	0.38 ± 0.03 ^d^	0.77 ± 0.11 ^d^	2.61 ± 0.17 ^c^	3.04 ± 0.24 ^c^
0.1 mg/L BPA	0.96 ± 0.10 ^a^	1.85 ± 0.27 ^a^	3.86 ± 0.42 ^a^	0.64 ± 0.11 ^a^	1.55 ± 1.06 ^a^	5.06 ± 0.94 ^a^	7.29 ± 1.52 ^a^
1 mg/L BPA	0.62 ± 0.05 ^d^	0.59 ± 0.12 ^c^	2.78 ± 0.55 ^b^	0.51 ± 0.09 ^b^	0.93 ± 0.12 ^c^	3.12 ± 1.01 ^b^	3.83 ± 0.84 ^c^
0.1 mg/L DEET	0.71 ± 0.21 ^c^	0.69 ± 0.11 ^b^	2.52 ± 0.09 ^b^	0.47 ± 0.05 ^c^	0.81 ± 0.24 ^c^	2.76 ± 0.11 ^c^	3.15 ± 0.08 ^c^
1 mg/L DEET	0.82 ± 0.16 ^b^	0.55 ± 0.07 ^c^	3.66 ± 0.28 ^a^	0.53 ± 0.15 ^b^	1.22 ± 0.19 ^b^	3.31 ± 0.32 ^b^	4.03 ± 0.25 ^b^
0.1 mg/L TRC	0.59 ± 0.12 ^d^	0.51 ± 0.11 ^c^	2.17 ± 0.18 ^b^	0.32 ± 0.09 ^d^	0.72 ± 0.05 ^d^	2.44 ± 0.13 ^c^	2.98 ± 0.16 ^c^
1 mg/L TRC	0.42 ± 0.10 ^e^	0.39 ± 0.14 ^d^	1.88 ± 0.15 ^c^	0.28 ± 0.11 ^e^	0.45 ± 0.16 ^e^	1.43 ± 0.23 ^d^	2.10 ± 0.30 ^d^
0.1 mg/L BPH	0.59 ± 0.14 ^d^	0.50 ± 0.02 ^c^	2.21 ± 0.16 ^b^	0.29 ± 0.08 ^e^	0.69 ± 0.22 ^d^	2.55 ± 0.25 ^c^	2.99 ± 0.12 ^c^
1 mg/L BPH	0.40 ± 0.19 ^e^	0.39 ± 0.21 ^d^	2.18 ± 0.12 ^b^	0.31 ± 0.14 ^d^	0.65 ± 0.10 ^d^	2.05 ± 0.38 ^c^	2.34 ± 0.33 ^cd^
0.1 mg/L α-END	0.45 ± 0.11 ^e^	0.41 ± 0.05 ^d^	2.00 ± 0.08 ^bc^	0.18 ± 0.11 ^f^	0.39 ± 0.15 ^f^	1.77 ± 0.25 ^d^	2.11 ± 0.26 ^d^
1 mg/L α-END	0.40 ± 0.07 ^e^	0.38 ± 0.07 ^d^	1.92 ± 0.16 ^c^	0.13 ± 0.01 ^f^	0.33 ± 0.07 ^f^	1.74 ± 0.16 ^d^	2.03 ± 0.15 ^d^
0.1 mg/L β-END	0.43 ± 0.05 ^e^	0.33 ± 0.02 ^e^	1.90 ± 0.11 ^c^	0.11 ± 0.05 ^f^	0.28 ± 0.03 ^f^	1.69 ± 0.14 ^d^	1.88 ± 0.22 ^d^
1 mg/L β-END	0.38 ± 0.06 ^e^	0.32 ± 0.04 ^e^	1.78 ± 0.08 ^c^	0.10 ± 0.03 ^f^	0.25 ± 0.01 ^f^	1.58 ± 0.11 ^d^	1.82 ± 0.08 ^d^
Mixture	0.56 ± 0.23 ^d^	0.46 ± 0.18 ^c^	2.01 ± 0.31 ^bc^	0.25 ± 0.41 ^e^	0.63 ± 0.20 ^d^	2.12 ± 0.33 ^c^	2.35 ± 0.26 ^cd^

**Table 3:** The comparison of the effect of different OMPs in *W. arrhiza* cultures in relation to the control on the 7th day of cultivation. The following symbols are used: ↑ means an increase in relation to the control; ↓ means a decrease in relation to the control; ≈ means no statistically significant influence in relation to the control.

**Biological Parameter**	**Treatment**						
	**BPA**	**DEET**	**TRC**	**BPH**	**α-END**	**β-END**	**Mixture**
Plant growth	↓	↓	↓	↓	↓	↓	↓
Chlorophyll *a*	↑	↑	↓	↑	↓	↓	↓
Chlorophyll *b*	↓	↓	↓	↓	↓	↓	↓
Carotenes	↑	↑	↓	↓	↓	↓	↓
Xanthophylls	↑	↑	↓	↓	↓	↓	↓
Proteins	↓	↓	↓	↓	↓	↓	↓
Monosaccharides	≈	↑	≈	↑	↑	↑	≈
MDA	↑	≈	↑	≈	≈	↑	↑
H_2_O_2_	↑	≈	↑	≈	≈	↑	↑

## Data Availability

The raw data supporting the conclusions of this article will be made available by the authors on request.
